# A cross-sectional study of the prevalence and associations of iron deficiency in a cohort of patients with chronic obstructive pulmonary disease

**DOI:** 10.1136/bmjopen-2015-007911

**Published:** 2015-07-01

**Authors:** Annabel H Nickol, Matthew C Frise, Hung-Yuan Cheng, Anne McGahey, Bethan M McFadyen, Tara Harris-Wright, Nicole K Bart, M Kate Curtis, Shivani Khandwala, David P O'Neill, Karen A Pollard, F Maxine Hardinge, Najib M Rahman, Andrew E Armitage, Keith L Dorrington, Hal Drakesmith, Peter J Ratcliffe, Peter A Robbins

**Affiliations:** 1 Oxford Centre for Respiratory Medicine and the Oxford Respiratory Trials Unit, Oxford University Hospitals NHS Trust, Churchill Hospital, Oxford, UK; 2 Department of Physiology, Anatomy and Genetics, University of Oxford, Oxford, UK; 3 Weatherall Institute of Molecular Medicine, University of Oxford, John Radcliffe Hospital, Oxford, UK; 4 Nuffield Department of Medicine, University of Oxford, Oxford, UK

**Keywords:** EPIDEMIOLOGY

## Abstract

**Objectives:**

Chronic obstructive pulmonary disease (COPD) is a major cause of morbidity and mortality. Iron deficiency, with or without anaemia, is associated with other chronic conditions, such as congestive heart failure, where it predicts a worse outcome. However, the prevalence of iron deficiency in COPD is unknown. This observational study aimed to determine the prevalence of iron deficiency in COPD and associations with differences in clinical phenotype.

**Setting:**

University hospital outpatient clinic.

**Participants:**

113 adult patients (65% male) with COPD diagnosed according to GOLD criteria (forced expiratory volume in 1 s (FEV_1_): forced vital capacity (FVC) ratio <0·70 and FEV_1_ <80% predicted); with age-matched and sex-matched control group consisting of 57 healthy individuals.

**Main outcome measures:**

Prevalence of iron deficiency, defined as: any one or more of (1) soluble transferrin receptor >28.1 nmol/L; (2) transferrin saturation <16% and (3) ferritin <12 µg/L. Severity of hypoxaemia, including resting peripheral arterial oxygen saturation (SpO_2_) and nocturnal oximetry; C reactive protein (CRP); FEV_1_; self-reported exacerbation rate and Shuttle Walk Test performance.

**Results:**

Iron deficiency was more common in patients with COPD (18%) compared with controls (5%). In the COPD cohort, CRP was higher in patients with iron deficiency (median 10.5 vs 4.0 mg/L, p<0.001), who were also more hypoxaemic than their iron-replete counterparts (median resting SpO_2_ 92% vs 95%, p<0.001), but haemoglobin concentration did not differ. Patients with iron deficiency had more self-reported exacerbations and a trend towards worse exercise tolerance.

**Conclusions:**

Non-anaemic iron deficiency is common in COPD and appears to be driven by inflammation. Iron deficiency associates with hypoxaemia, an excess of exacerbations and, possibly, worse exercise tolerance, all markers of poor prognosis. Given that it has been shown to be beneficial in other chronic diseases, intravenous iron therapy should be explored as a novel therapeutic option in COPD.

**Strengths and limitations of this study**

- The patients who took part in the study were comprehensively evaluated and had disease severity assessed according to a variety of well-validated measures, many known to predict outcome in chronic obstructive pulmonary disease (COPD).
- The definition of iron deficiency was conservative and based on several different validated indices.
- The study cohort was of limited size compared to other COPD cohorts.
- The patient cohort was almost exclusively Caucasian with moderately severe COPD; the findings may not apply to other ethnic groups or those with different disease severity.

## Introduction

The effects of iron deficiency on haemoglobin concentration are well known, but less well recognised are the earlier consequences prior to the development of anaemia. In otherwise healthy individuals, these include reduced aerobic exercise capacity, higher levels of fatigue and impaired cognition.^1^ ^2^ Iron deficiency is common in patients with congestive heart failure, where it has been identified as an independent predictor of mortality.^3^ In this setting, it has been shown that treatment with intravenous iron improves functional outcomes regardless of the presence or absence of anaemia, and regardless of whether or not haemoglobin concentrations change following iron therapy.^4^ ^5^

Iron has a pivotal role in the pathways that cells use to sense and respond to hypoxia,^6^ with iron deficiency to an extent mimicking hypoxia. This may underlie some of the symptomatology associated with iron deficiency.^7^ A fall in alveolar oxygen tension causes hypoxic pulmonary vasoconstriction (HPV), and chronic hypoxia can lead to irreversible remodelling of the pulmonary vasculature and pulmonary hypertension. The influence of iron on HPV is demonstrated by the striking effects of experimental manipulation of iron levels in healthy humans. Iron depletion augments the pulmonary hypertensive response to hypoxia, while iron loading greatly attenuates the phenomenon.^8^ ^9^

In chronic obstructive pulmonary disease (COPD), iron deficiency could be particularly deleterious since hypoxaemia is common, is a marker of disease severity, and is important in the pathophysiology and extrapulmonary manifestations of the condition.^10^ Pulmonary hypertension is one of the strongest predictors of decreased survival in COPD and is significantly driven by hypoxia;^11^ it may also be augmented by iron deficiency. However, the prevalence, aetiology and pathophysiology of iron deficiency in the setting of COPD are unknown.

In this study, we examined iron status in the Oxford Biomedical Research Centre (BRC) COPD Cohort. We used the traditional laboratory measures of ferritin and transferrin saturation (TSat) as well as newer markers, such as hepcidin and soluble transferrin receptor (sTfR). The peptide hormone hepcidin is now understood to be central to iron homoeostasis but is also strongly influenced by the innate immune system and erythropoietic drive. Inflammation elevates hepcidin, which reduces serum iron and dietary iron absorption.^12^ Hepcidin is therefore important in the pathogenesis of the anaemia of chronic disease.^13^ Measurement of sTfR has emerged as a tool to distinguish between pure iron-deficiency anaemia and the anaemia of chronic disease.^14^ We also measured erythropoietin (EPO), C reactive protein (CRP) and functional markers—patient-reported outcome measures and walking distance. The relationship between iron status and these variables was explored.

## Methods

### Patient selection criteria

The Oxford BRC COPD Cohort comprises a carefully phenotyped group of patients with a clinical diagnosis of COPD made according to GOLD criteria^15^ (forced expiratory volume in 1 s (FEV_1_): forced vital capacity (FVC) ratio <0·70 and FEV_1_ <80% predicted) using standard predicted values.^16^ Criteria for enrolment included the absence of other significant cardiopulmonary disease or comorbidity likely to limit life-expectancy below 2 years. It was estimated that a sample of just over 100 patients would be needed to demonstrate a 20% prevalence of iron deficiency with 80% confidence and 5% precision, assuming an effectively unlimited population. Between August 2009 and April 2012, patients attending a specialist COPD clinic at a university hospital were invited to enrol in the cohort. The study was conducted in accordance with the Declaration of Helsinki and approval was given by the NHS South Central Berkshire Research Ethics Committee. Written informed consent was given by all participants.

### Control cohort

A group of healthy individuals without evidence of any acute illness, who had been recruited for a different study examining iron and exercise physiology (approved by the NHS South Central Oxford B Research Ethics Committee), provided control data for iron status. Although this group was similar in size to the COPD cohort, there was a greater proportion of females and the average age was younger than for the COPD cohort. In order to overcome these differences, a computer algorithm was used to match cases with controls in a 2:1 ratio, and obtain a control cohort that was well matched for sex and age.

### Study design and procedures

This was an observational study in patients having stable disease when enrolled. Assessments were undertaken when patients reported being exacerbation-free for at least 4 weeks and included history, record of exacerbations, clinical examination and spirometry. Resting pulse oximetry and arterial or capillary blood gas analysis were performed, and nocturnal mean arterial oxygen saturation and proportion of time spent asleep with SpO_2_ <90% were also determined (Konica Minolta Pulsox 300i; Stowood Scientific, UK). The St George's Respiratory Questionnaire (SGRQ),^17^ Hospital Anxiety and Depression (HAD) score^18^ and Epworth Sleepiness Score (ESS)^19^ were also used. All patients underwent two Shuttle Walk Tests (SWT),^20^ with the better result used for analysis. Whole blood was obtained from patients at the same visit. Full blood count, serum iron, TSat, ferritin and CRP were measured at a central laboratory. Serum and plasma were obtained by centrifugation and immediately frozen at −80°C for subsequent analysis. sTfR and EPO were measured by ELISA (Quantikine, R&D Systems, Abingdon, UK) as was hepcidin (Hepcidin-25 EIA Kit, Bachem, Peninsula Laboratories, San Carlos, California, USA) in accordance with manufacturers’ specifications.

### Definition of iron deficiency

Iron deficiency was defined as any one or more of (1) sTfR >28.1 nmol/L; (2) TSat <16% and (3) serum ferritin <12 µg/L. Patients were classified as iron replete (IR) if they were not iron deficient (ID) and had TSat ≥20%. Patients who did not meet the definition of iron deficiency but who had 16% ≤ TSat <20% were viewed as falling into an indeterminate group. The rationale for this definition was as follows: in clinical practice, measurement of serum ferritin is central to the assessment of iron status with a serum ferritin level <12 µg/L having a very high positive predictive value for absolute iron deficiency.^21^ ^22^ As an acute-phase reactant, however, its utility is limited in the presence of inflammation. Therefore, while low serum ferritin is diagnostic of iron deficiency, normal serum ferritin cannot be used to exclude it. The transferrin receptor allows cells to take up iron and a proportion can be detected in a freely circulating form, sTfR. Inflammation does not significantly affect sTfR levels, which reflect total transferrin receptor expression and thus unmet iron requirements. The sTfR cut-off of 28.1 nmol/L is based on the upper reference limit (mean plus two SDs) of the assay when used in healthy sea-level Caucasian patients.^14^ A TSat ≥20% is associated with normal red cell indices, while a value below 16% indicates an insufficient supply of iron to the erythroid marrow,^23^ which will eventually manifest as a microcytic, hypochromic anaemia.

### Data analysis

Data were analysed and graphs plotted using SPSS (V.20, IBM) and SigmaPlot (V.12.3, Systat Software). Non-parametric data comparisons were made using the Mann-Whitney U (MWU) test and parametric data analysed using a two-sided Student t test. To investigate the effect of possible confounders, analysis of covariance (ANCOVA) was used. Spearman's rank correlation coefficient was calculated for analysis of correlation between iron status rank (treating sTfR and TSat of equal importance) and non-parametric variables. The algorithm used to generate the matched control cohort was written in MATLAB (MathWorks).

## Results

One hundred and sixteen patients were enrolled in the COPD cohort, with complete laboratory data available for 113 patients (74 men, 39 women). The cohort was thus 65% male with a mean (SD) age of 67.6 (8.77) years. Patients had moderately severe COPD as evidenced by a median FEV_1_ of 41% predicted, with a median pack-year history of 43.0, the majority being ex-smokers. All but two of the patients were Caucasians. Five patients were receiving long-term oxygen therapy, and nine more had access to short-burst home oxygen (supplemental oxygen for intermittent use to relieve severe dyspnoea). The control cohort consisted of 57 individuals, 37 (65%) of whom were male, with a mean (SD) age of 67.6 (6.10) years. All were Caucasians.

### Iron deficiency is common in COPD

**Figure 1:**
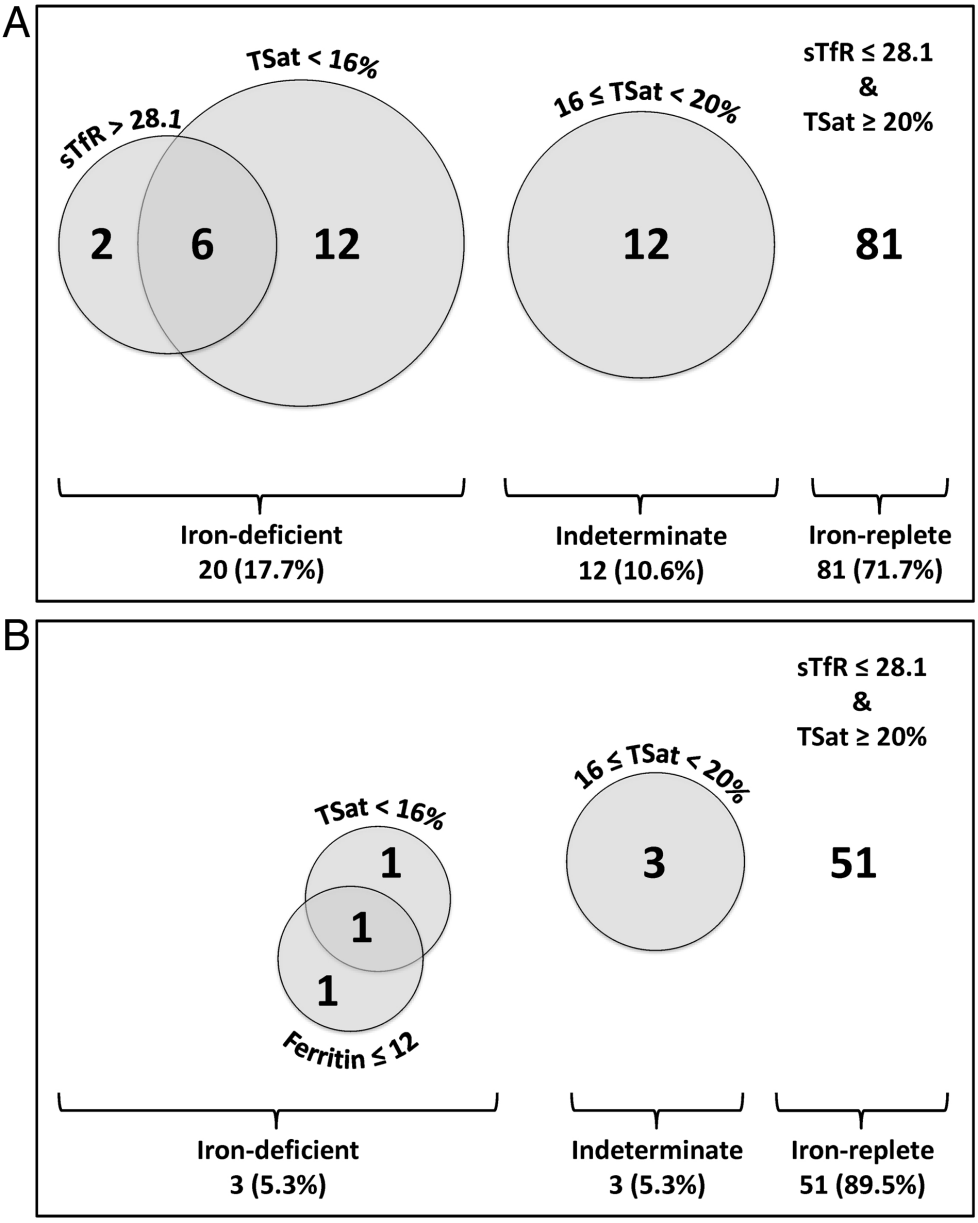
(A) Venn diagram showing proportions of patients with COPD who were iron deficient, of indeterminate iron status or iron replete. No patients with COPD had a serum ferritin <12 µg/L. (B) Corresponding diagram for healthy control cohort. No healthy control participant had an sTfR >28.1 nmol/L (COPD, chronic obstructive pulmonary disease; sTfR, soluble transferrin receptor; TSat, transferrin saturation).

Twenty patients with COPD (17.7%) were ID, 81 (71.7%) IR and 12 (10.6%) indeterminate (figure 1A). This contrasts with 3 (5.3%), 51 (89.5%) and 3 (5.3%), respectively, for the control cohort (p=0.029, χ^2^) (figure 1B). For the COPD cohort, there were no differences between the ID and IR groups in sex, smoking status, median number of pack years smoked or proportion receiving oxygen therapy. FEV_1_ appeared to be lower in the ID group but this difference was not statistically significant. Equally, in the COPD cohort as a whole, there was not a significant correlation between FEV_1_ and any index of iron status, be it sTfR, TSat or overall iron status rank (r=−0.068, p=0.477; r=0.111, p=0.240 and r=0.083, p=0.384; respectively). Characteristics of the COPD cohort are given in table 1.

**Table 1:** COPD cohort patient characteristics

	All patients (113)	Iron deficient (20)	Iron replete (81)	Statistical comparison
Male sex; n (%)	74 (65)	12 (60)	53 (66)	χ^2^ (p=0.793)
Age, years; mean (SD)	67.6 (8.77)	67.5 (9.86)	67.5 (8.47)	t test (p=0.980)
FEV_1_, % predicted; mean (SD)	43.8 (17.1)	39.7 (16.5)	44.8 (17.0)	t test (p=0.236)
Current smoker; n (%)	17 (15)	4 (20)	11 (13.6)	χ^2^ (p=0.710)
Pack years; median (IQR)	44.0 (28.5–62.0)	44.0 (37.0–67.0)	45.0 (27.8–62.8)	MWU test (p=0.799)
BMI, kg/m^2^; median (IQR)	27.6 (21.7–30.3)	27.8 (21.6–31.9)	27.5 (21.7–30.6)	MWU test (p=0.855)
Long-term oxygen therapy; n (%)	5 (4)	2 (10)	3 (4)	χ^2^ (p=0.531)
Any home oxygen therapy; n (%)	14 (12)	5 (25)	8 (10)	χ^2^ (p=0.151)

BMI, body mass index; COPD, chronic obstructive pulmonary disease; FEV, forced expiratory volume in 1 s; MWU, Mann-Whitney U.

### Inflammation is prevalent in COPD and associates with iron deficiency

**Figure 2:**
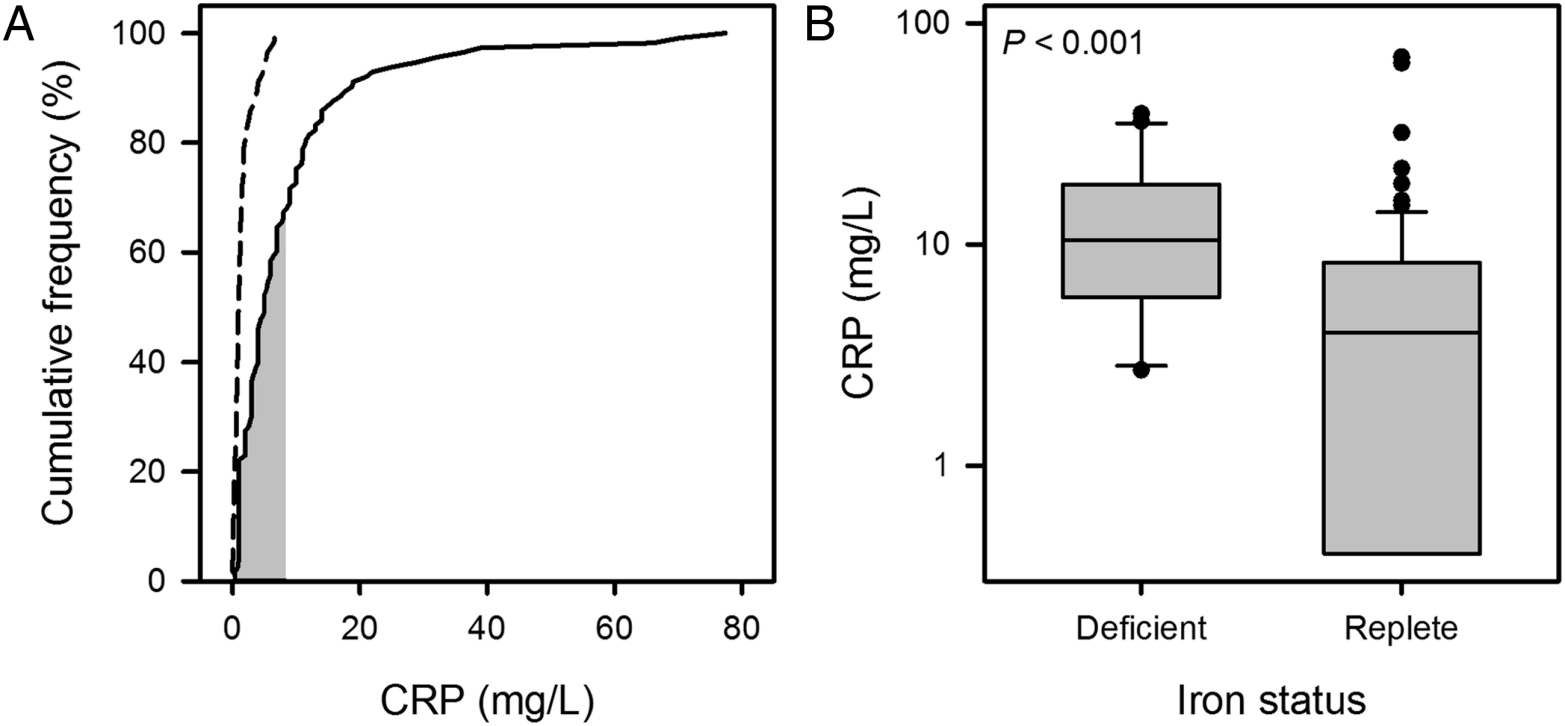
(A) Cumulative frequency plot for C reactive protein (CRP). Data for the chronic obstructive pulmonary disease (COPD) cohort are plotted with a solid line and those for the control cohort with a dashed line; the shaded area indicates the normal range for the assay. (B) Box plot (boxes show IQR and median, whiskers show 10th and 90th centiles, circles are outliers) showing distribution of results for CRP by iron status in the COPD cohort. CRP was significantly higher in the iron-deficient (ID) group (median 10.5 vs 4.0 mg/L, p<0.001).

In the COPD cohort, 37 patients (33%) had a CRP greater than 8 mg/L, the upper limit of normal for the assay used (figure 2A). In contrast, there was none in the control cohort (p<0.001, χ^2^). For the patients with COPD, CRP was significantly higher in the ID than in the IR group (median 10.5 vs 4.0, p<0.001; figure 2B), a relationship that persisted after adjustment for differences in FEV_1_ (p=0.028). The ID group had a higher self-reported rate of exacerbations in the year prior to enrolment (median 3 (IQR 2–6) vs 2 (IQR 1–4); p=0.024). Again, this relationship persisted after adjustment for differences in FEV_1_ (p=0.045).

### Iron status and inflammation influence ferritin and hepcidin levels

**Figure 3:**
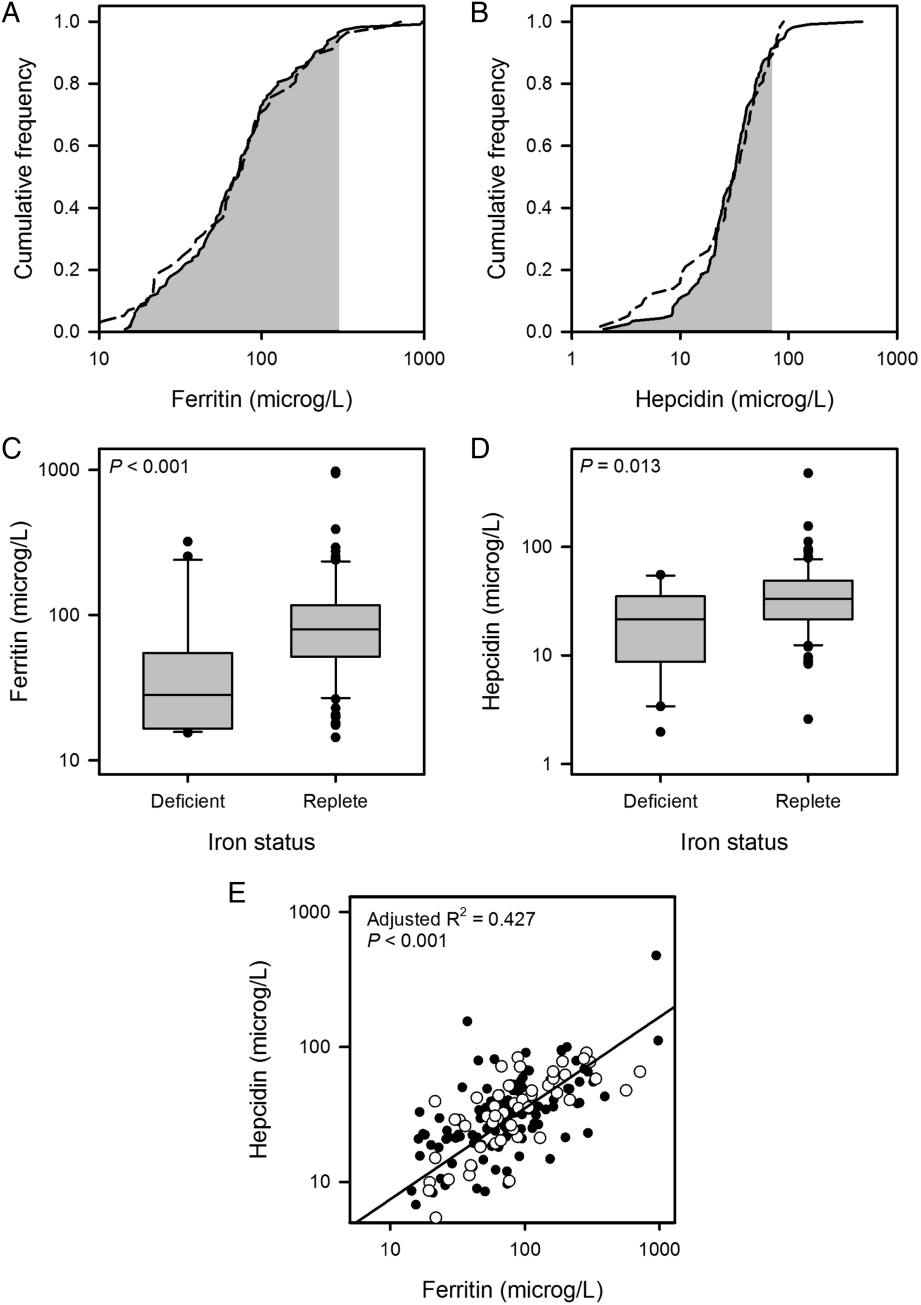
(A and B) Cumulative frequency plots for ferritin and hepcidin. Data for the chronic obstructive pulmonary disease (COPD) cohort are plotted with a solid line and those for the control cohort with a dashed line; the shaded area indicates the normal range for each assay. (C and D) Box plots showing distribution of results for ferritin and hepcidin by iron status in the COPD cohort. Ferritin (median 28.3 vs 79.9 µg/L; p<0.001) and hepcidin (median 21.4 vs 33.4 µg/L; p=0.013) were both lower in the iron-deficient (ID) group. (E) Scatter plot showing relationship between hepcidin and ferritin in the COPD cohort (filled circles) and control cohort (empty circles); the regression line is for both groups taken together; individual regression lines were not significantly different.

Figure 3 shows cumulative frequency plots for ferritin and hepcidin. These were similar for COPD and control cohorts. A positive correlation between hepcidin and ferritin values was seen for patients with COPD as well as for healthy controls (figure 3E). For both COPD and control cohorts, ferritin and hepcidin levels were significantly lower in ID versus IR individuals (COPD cohort medians: ferritin 28.3 vs 79.9 µg/L, p<0.001; hepcidin 21.4 vs 33.4 µg/L, p=0.013; control cohort medians: ferritin 10.5 vs 76.3 µg/L, p=0.008; hepcidin 3.33 vs 35.1 µg/L, p=0.007). For the COPD cohort, linear regression analysis indicated CRP was a significant factor determining both ferritin (p<0.001) and hepcidin (p<0.001). This was not the case for the healthy control cohort, where CRP values were low. Consistent with the elevation of ferritin and hepcidin by inflammation, the values for these were significantly higher in the COPD ID subgroup compared with the control ID subgroup (median ferritin 28.3 vs 10.5 µg/L, p=0.040; median hepcidin 21.4 vs 3.33 µg/L, p=0.020).

### Iron deficiency is associated with severity of hypoxaemia

**Figure 4:**
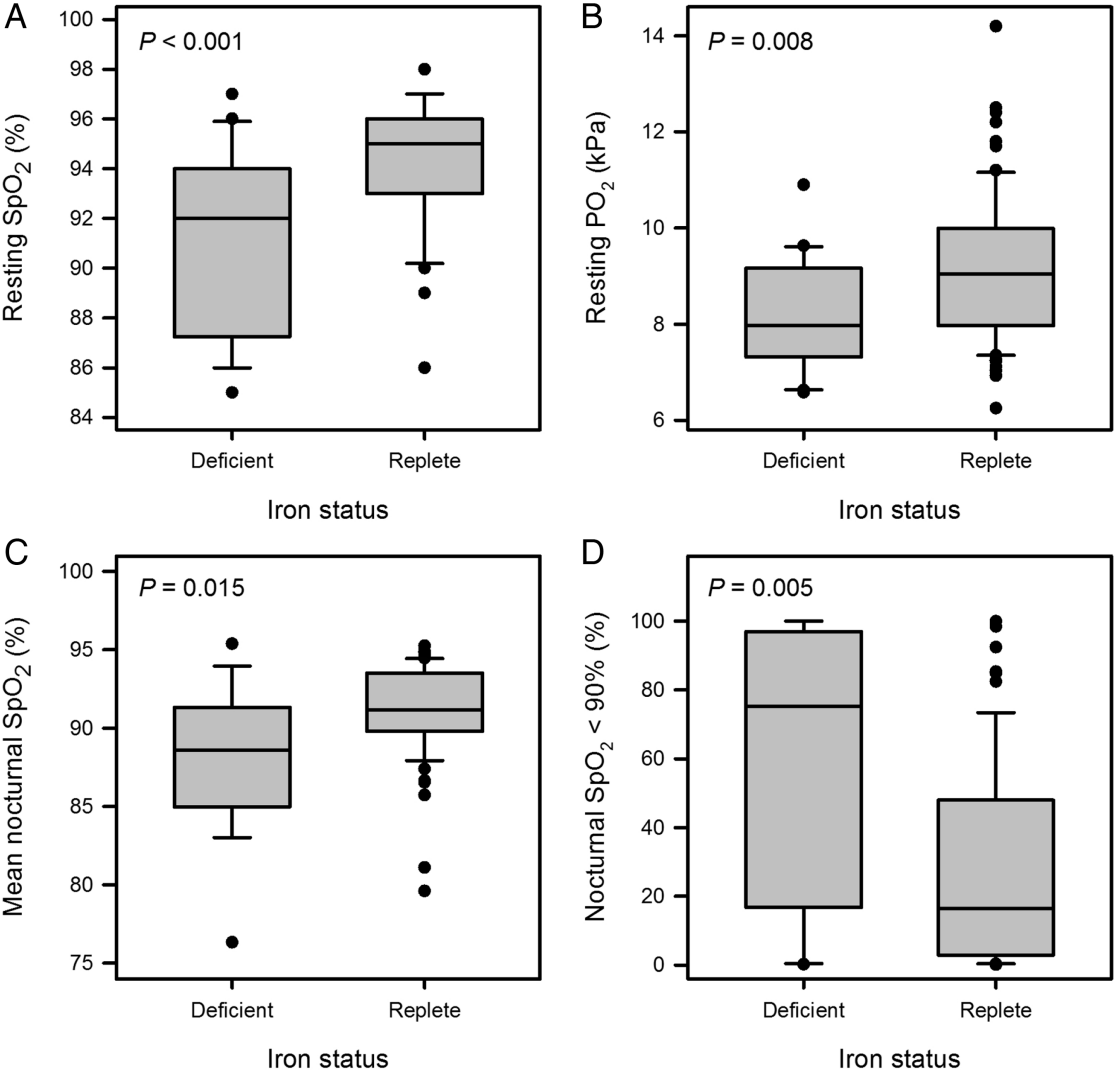
Box plots showing distribution of (A) resting peripheral oxygen saturation (SpO_2_), (B) resting PO_2_ on blood gas, (C) mean nocturnal SpO_2_ and (D) percentage of nocturnal SpO_2_ recordings <90%, all by iron status, in the chronic obstructive pulmonary disease (COPD) cohort. All measures were significantly worse in the iron-deficient (ID) group (medians 92 vs 95%, p<0.001; 7.97 vs 9.05 kPa, p=0.008; 88.6 vs 91.2%, p=0.015; and 75.3 vs 16.4%; p=0.005, respectively).

Within the COPD cohort, resting daytime SpO_2_ was significantly lower in the ID group, as were PO_2_ on a capillary or arterial blood gas sample and mean nocturnal SpO_2_. Additionally, the proportion of time spent with nocturnal SpO_2_ <90% was much higher in the ID group. Figure 4 illustrates these findings. All these relationships persisted after adjustment for FEV_1_ (p<0.001, p=0.006, p=0.001 and p=0.001, respectively).

### Haemoglobin concentration does not differ according to iron status in COPD

**Figure 5:**
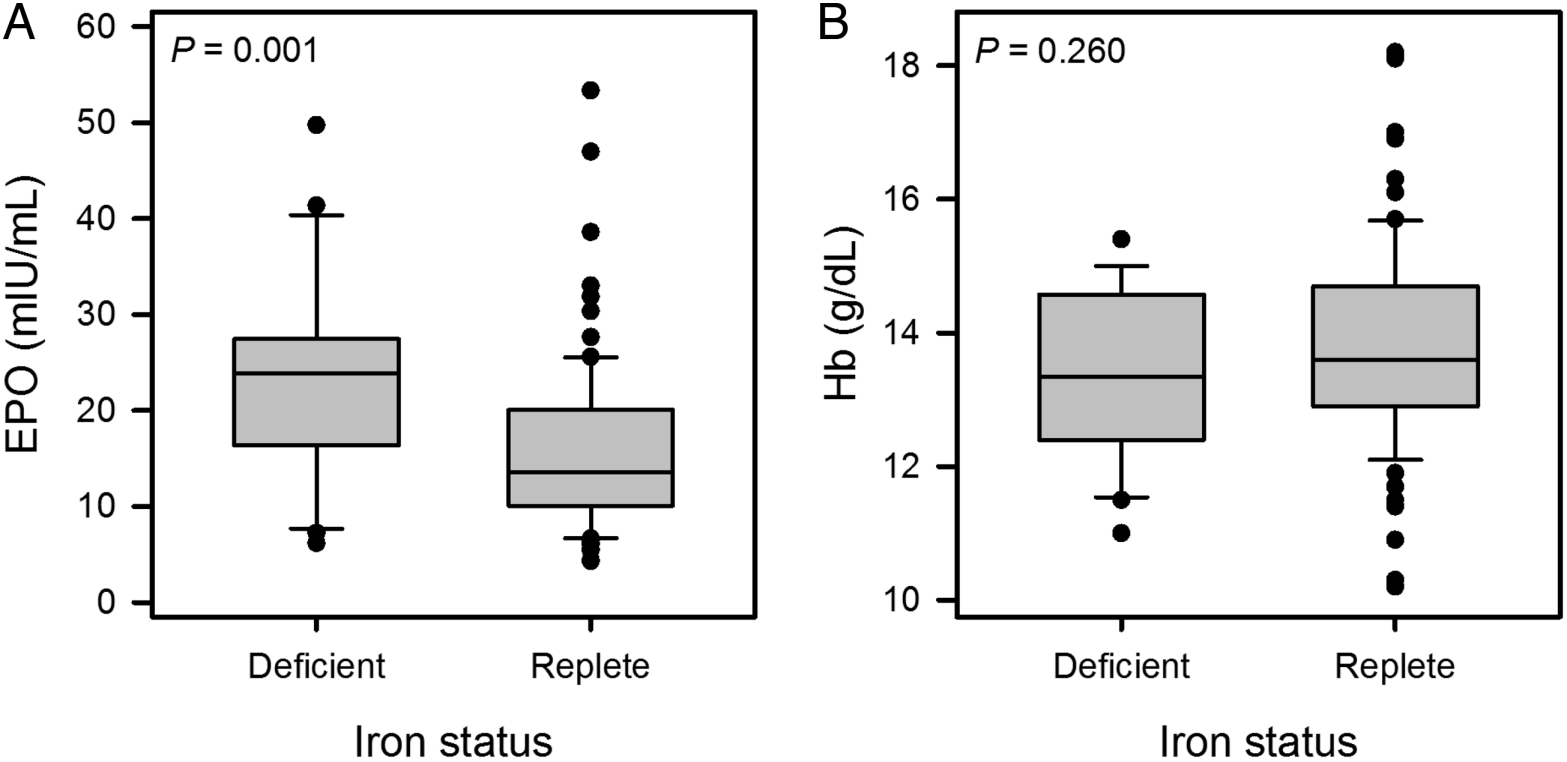
Box plots showing distribution of (A) erythropoietin (EPO) and (B) haemoglobin (Hb) concentration, by iron status in the chronic obstructive pulmonary disease (COPD) cohort. EPO was significantly higher in the iron-deficient (ID) group (median 23.9 vs 13.5 mIU/mL, p=0.001) but Hb did not differ (mean 13.4 vs 13.8 g/dL; p=0.260).

Haemoglobin levels were not significantly different in patients with COPD between ID and IR groups despite a significantly higher EPO level in the ID group (figure 5). However, the mean cell volume (MCV) was lower in the ID group (mean 88.8 vs 93.1 fL; p=0.001).

### Iron status and functional performance

**Figure 6:**
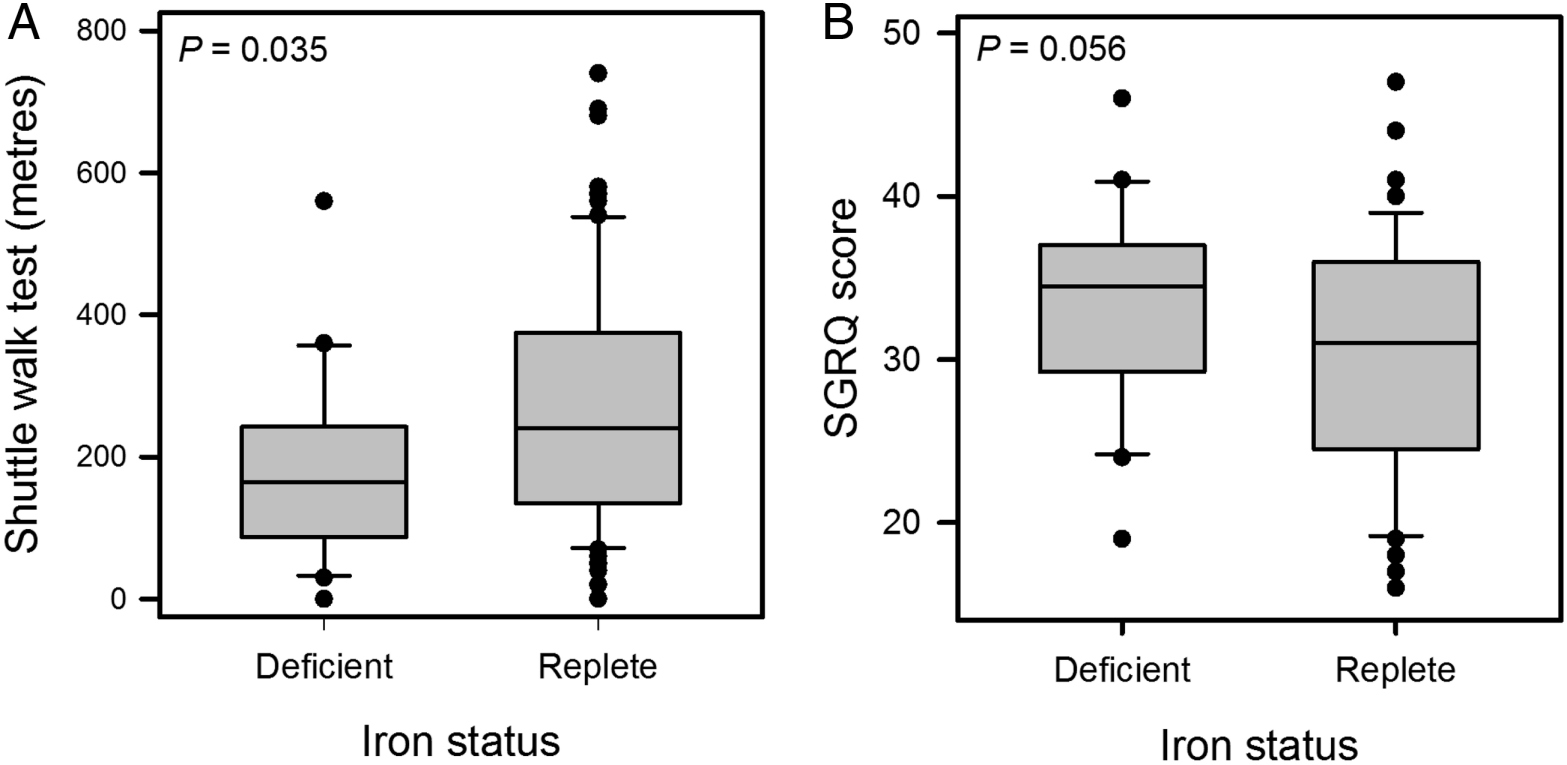
Box plots showing distribution of (A) Shuttle Walk Test (SWT) distance and (B) St George's Respiratory Questionnaire (SGRQ) score, by iron status in the chronic obstructive pulmonary disease (COPD) cohort. SWT distance was lower in the iron-deficient (ID) group (median 165 vs 240 m, p=0.035) although statistical significance was lost after correction for difference in FEV_1_ (p=0.081). The difference in SGRQ scores was not statistically significant (mean 33.5 vs 30.1, p=0.056).

Exercise ability, as measured by the SWT, was worse in the ID group (median 165 vs 240 m, p=0.035; figure 6) but statistical significance was lost after adjusting for differences in FEV_1_ (p=0.081). Differences in SGRQ scores, HAD scores, and ESS between ID and IR groups, did not reach statistical significance (mean 33.5 vs 30.1, p=0.056; median 13.5 vs 11, p=0.133 and median 8 vs 5, p=0.279, respectively).

## Discussion

This study has demonstrated a high prevalence of non-anaemic iron deficiency in COPD that may be driven by inflammation. Patients with iron deficiency were more hypoxaemic even though they did not have significantly worse airflow limitation. Such marked daytime and nocturnal hypoxaemia in the ID group was unexpected. One possible explanation arises from the essential role of iron as a cofactor in a key cellular pathway that senses hypoxia, and modulates levels of the hypoxia-inducible factor family of transcription factors.^6^ ^8^ ^9^ Tissue iron deficiency alters the effect of hypoxia on the pulmonary vasculature,^8^ and thus may impair physiological ventilation to perfusion matching and worsen hypoxaemia. Irrespective of the underlying mechanisms, the high prevalence of iron deficiency in COPD and the newly identified association of iron deficiency with hypoxaemia are potentially of considerable clinical significance.

The major hormone that acts to determine iron availability and distribution is hepcidin. Serum levels of hepcidin are influenced by iron, hypoxia, erythropoietic drive and inflammation.^12^ The importance of inflammation in regulating iron availability in COPD is illustrated by the failure of the combination of iron deficiency and hypoxia to suppress hepcidin completely in the ID COPD group, whereas it is barely detectable in the ID healthy controls. This ‘inappropriate expression’ of hepcidin is similar to that seen in the anaemia of chronic disease.^24^

Within the ID COPD group, despite significantly lower ferritin and MCV, anaemia was uncommon and mean haemoglobin concentration was not lower than in the IR patients. The ID group had higher EPO levels, consistent with appropriate sensing of hypoxaemia, but a failure of the marrow to respond accordingly. It appears as a tension exists here between erythropoietic drive and iron availability, with inflammation-driven, hepcidin-mediated iron sequestration constraining a rise in haemoglobin. In support of this suggestion, it was noted 50 years ago that individuals with severe lung disease tended to increment their haemoglobin only if given intramuscular iron.^25^

The findings in the present study have parallels with those from studies exploring iron homoeostasis in other chronic respiratory diseases. In pulmonary vascular disease, iron deficiency is common.^26–28^ In patients with idiopathic pulmonary arterial hypertension, the prevalence of non-anaemic iron deficiency approaches two-thirds, and severity of iron deficiency correlates with WHO functional class and worsening exercise capacity, independent of haemoglobin concentration.^29^ Recently, iron deficiency has been shown to be associated with worse lung function, and higher iron stores negatively correlated with risk of asthma, in a large cohort of north American women.^30^ Whether intravenous iron therapy may be beneficial for patients with idiopathic pulmonary arterial hypertension is the subject of a large ongoing randomised-controlled clinical trial,^31^ and a small pilot study has recently described short-term improvement in functional outcomes with intravenous iron in this setting, though no data on pulmonary artery pressure were reported.^32^ With respect to COPD, pulmonary hypertension^11^ and hypoxaemia^10^ are both predictors of mortality, and therefore iron deficiency and hypoxaemia may interact in a particularly deleterious way in this condition.

**Figure 7:**
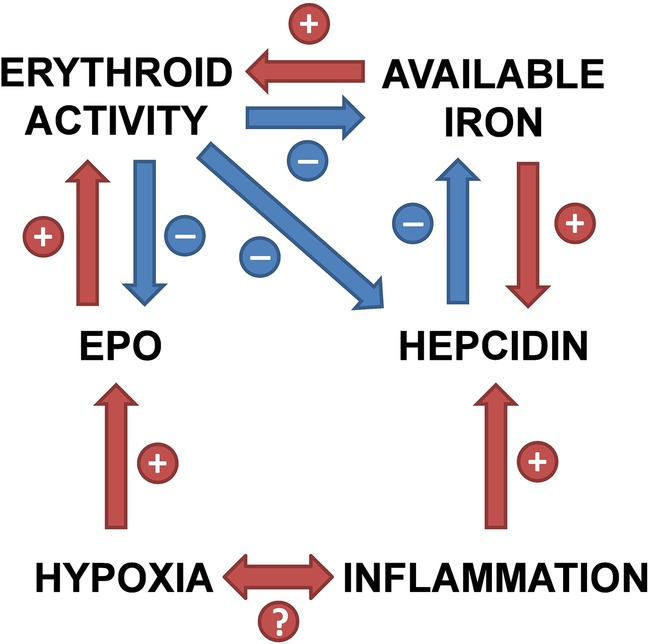
Relationships between hypoxia, inflammation and iron homoeostasis, mediated by hepcidin (EPO, erythropoietin).

In adult patients with another chronic inflammatory respiratory disorder, cystic fibrosis, an impaired erythropoietic response to hypoxaemia has also been described,^33^ apparently associated with inflammation, as in our COPD cohort. In a separate study, a 3-month course of oral iron was shown not to increase haemoglobin in a subgroup of patients with cystic fibrosis and functional iron deficiency (defined as TSat <16%).^34^ However, a very small case series of adult patients with cystic fibrosis given intravenous iron for anaemia refractory to oral iron found that haemoglobin concentration and MCV rose significantly within days of therapy.^35^ These findings are consistent with suppression of iron absorption by hepcidin. Figure 7 gives a schematic representation of the influence of hepcidin in the setting of chronic inflammation.

Non-invasive assessment of iron status is challenging in COPD, as in other chronic inflammatory conditions, since serum ferritin may be normal or raised despite inadequate iron availability. None of the patients with COPD had a serum ferritin level that would be considered strictly diagnostic of absolute iron deficiency. However, the lower MCV in the ID group argues that we did identify individuals with iron-restricted erythropoiesis. Importantly, the relationships observed between iron status, hypoxaemia and inflammation were essentially unchanged when additional analyses were performed using definitions of iron deficiency based either solely on low TSat, solely on raised sTfR, or on an even more restrictive requirement for both low TSat and low sTfR. We would therefore argue that, in this setting, each measure detects iron sequestration, the locking-away of iron such that it is unavailable for a particular physiological process,^13^ and thus functional iron deficiency.

The ID patients in our cohort had a significantly higher rate of self-reported exacerbations in the preceding year. This is important, since exacerbations contribute to COPD progression and strongly predict outcome.^36–38^ Furthermore, our study may suggest greater impairment of exercise capacity in ID patients with COPD, though statistical significance was lost after correction for differences in FEV_1_. When our COPD cohort was established, the SWT was chosen over the self-paced 6 min walk test because the performance has been reported to correlate strongly with maximum oxygen consumption,^39^ ^40^ which itself may be affected by iron deficiency. Numerically, the ID group managed only two-thirds the SWT distance of their IR counterparts. The minimum clinically important difference in the SWT, based on data from a cohort similar to ours undergoing a pulmonary rehabilitation programme, has been determined as 47.5 m, with patients reporting additional benefit at 78.7 m.^41^ Interestingly, this latter figure is approximately the difference between medians of the ID and IR groups. The SWT has been shown independently to predict survival in patients with COPD, with one study finding nearly a threefold higher mortality during an average of 4½ years of follow-up when SWT distance fell below 170 m,^42^ a value higher than the median distance achieved by our ID group. However, one of the limitations of the present study is that the size of our COPD cohort limits the power of functional comparisons between groups, so these data await validation in larger studies. A further limitation is that our patients were almost all Caucasian and had moderately severe COPD, so our findings may not apply to other ethnic groups or to those with disease of greater or lesser severity.

Recently, others have begun to consider the importance of disturbed iron homoeostasis in COPD, but anaemia continues to be a central focus.^43^ ^44^ It is certainly the case that, as in other chronic conditions, anaemia predicts a worse outcome in COPD,^45^ both in the setting of admission with an acute exacerbation^46^ and in the long term.^47^ ^48^ Taken as a whole, though, our findings suggest greater attention should be paid to iron deficiency irrespective of the presence or absence of anaemia. Novel therapeutic possibilities include both the manipulation of iron status through intravenous iron therapy and, tantalisingly, hepcidin antagonists becoming available.
